# Corrigendum: Enterococcus faecalis co-cultured with oral cancer cells exhibits higher virulence and promotes cancer cell survival, proliferation, and migration: an in vitro study

**DOI:** 10.1099/jmm.0.002141

**Published:** 2026-03-13

**Authors:** Fida Fathima, Yuvarajan Subramaniyan, Akshatha Rai, Punchappady Devasya Rekha

**Affiliations:** 1 Division of Microbiology and Biotechnology, Yenepoya Research Centre, Yenepoya (Deemed to be University), Mangalore, 575018, India

In the published version of the article [1], the incorrect control panel images were included in Fig. 3(a). The authors became aware of this due to a query raised on PubPeer [2], where it was noted that this figure had been used previously in another publication reporting on different experimental data [3].

The authors informed the publisher that the data presented in both published articles formed part of one of the author’s PhD thesis. The thesis used one representative image of the *Lactiplantibacillus plantarum* control to portray the various control experiments conducted during the PhD. As article [1] was in preparation at the same time, the incorrect control image was mistakenly used from the thesis to portray control experiments conducted for *Enterococcus faecalis*.

The authors provided their laboratory book records to support that separate control experiments were conducted for both the *E. faecalis* and *L. plantarum* studies. The authors also provided the original raw images for the *E. faecalis* experiments.

The authors are, therefore, replacing the control panel experiments in Fig. 3a with the following image from the correct experimental set:

**Figure:**
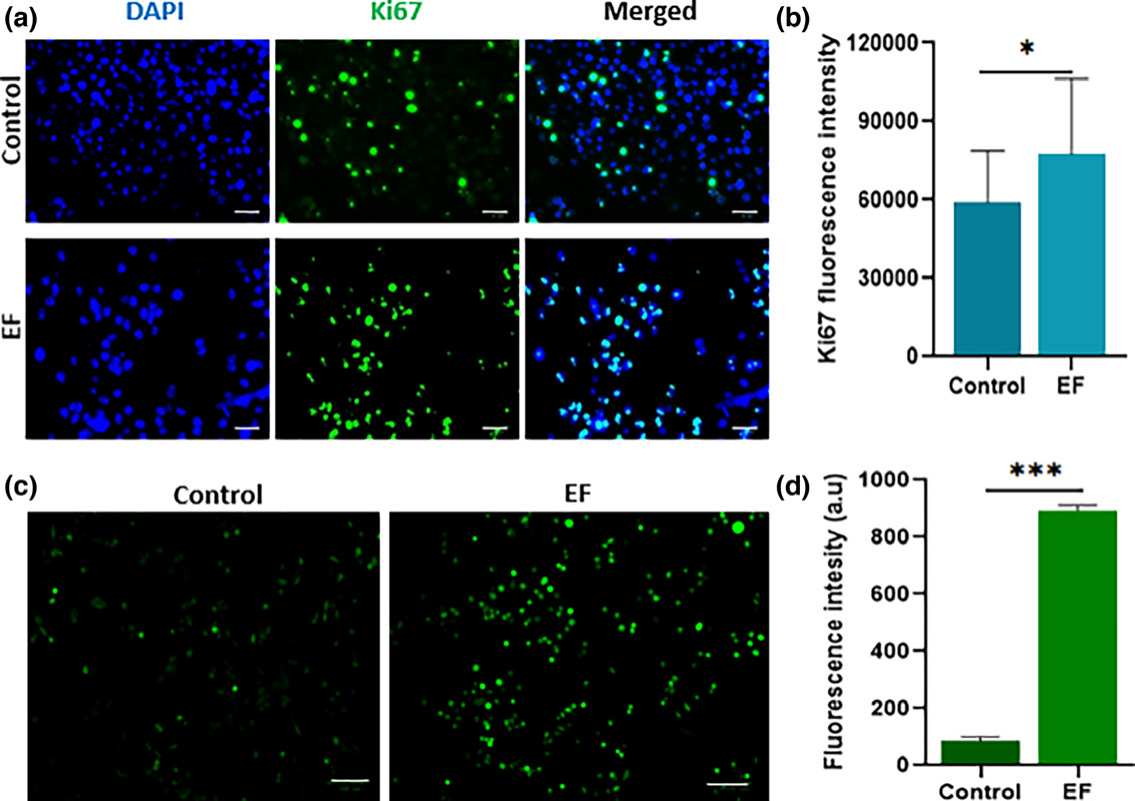
**Fig. 3** Ki67 expression and ROS production in Cal27 cells co-cultured with *E. faecalis*. (a) Fluorescence microscopic images and (b) quantitative measurement of Ki67 expression in Cal27 cells treated with *E. faecalis* (EF). The green colour indicates cells expressing Ki67, and the blue colour indicates nuclear staining using DAPI (scale bar=50 µm). (c) Fluorescence microscopic images and (d) fluorescence intensity of ROS generated in Cal27 cells co-cultured with *E. faecalis* (EF) at 6 h were detected using the DCFDA method and compared with untreated control. Data are presented as mean±sd (*n*=3). Statistical analysis was assessed using the Student’s *t*-test. *P<0.5 and ***P<0.001 (scale bar=100 µm).

The authors confirm that this corrected image does not affect the results or scientific conclusions of the article. The authors sincerely regret this error and take full responsibility for this oversight during all stages of the publication process.
